# Association between circulating resistin levels and thyroid dysfunction: A systematic review and meta-analysis

**DOI:** 10.3389/fendo.2022.1071922

**Published:** 2023-01-04

**Authors:** Lin Zhou, Kang Song, Wei Luo

**Affiliations:** ^1^ Department of Postgraduate, Qinghai University, Xining, China; ^2^ Department of Endocrinology, Qinghai Provincial People’s Hospital, Xining, China

**Keywords:** thyroid dysfunction, hyperthyroidism, hypothyroidism, resistin, meta-analysis

## Abstract

**Background:**

As a product of adipose tissue, resistin exceeds other adipokines in its role in regulating appetite, energy expenditure, insulin sensitivity, inflammation, and immunity, similar to thyroid hormones. This study aimed to evaluate the association between resistin levels and thyroid dysfunction and to explore variations in circulating resistin levels before and after treatment for thyroid dysfunction.

**Methods:**

This study was conducted according to the Preferred Reporting Items for Systematic Review and Meta-Analysis statement. A comprehensive search of PubMed, Embase, and Cochrane databases was conducted until June 15, 2022, with no start date restriction, according to the preregistered protocol (PROSPERO-CRD42022336617). RevMan version 5.4 and R software package version 4.2.0 were used for statistical analyses.

**Results:**

Fourteen studies with 1716 participants were included in this study. The findings of the meta-analysis confirmed that the resistin levels of patients with thyroid dysfunction were significantly higher than those of the euthyroid function control group (mean difference [MD] = 2.11, 95% confidence interval [CI] = 1.11–3.11, P < 0.00001). Furthermore, the resistin levels of patients with hyperthyroidism (MD = 3.23, 95% CI = 0.68–5.79, P = 0.01) and subclinical hypoidism (MD = 1.37, 95% CI = 0.31–2.42, P = 0.01) were significantly higher than those of euthyroid controls. The resistin levels of patients with thyroid dysfunction after treatment were significantly lower than those before treatment (MD = 1.00, 95% CI = 0.34–1.65, P = 0.003), especially in patients with hyperthyroidism (MD = 2.16, 95% CI = 1.00–3.32, P = 0.0003). Correlation analysis confirmed a positive correlation between resistin levels and free triiodothyronine (FT3) levels in patients with thyroid dysfunction (r = 0.27578, P = 0.001).

**Conclusions:**

Our meta-analysis demonstrates that resistin levels are significantly higher in patients with thyroid dysfunction, and the resistin levels after treatment in patients with thyroid dysfunction are significantly lower than those before treatment. Correlation analysis shows a positive correlation between resistin levels and FT3 levels in patients with thyroid dysfunction.

**Systematic review registration:**

https://www.crd.york.ac.uk/PROSPERO/, identifier CRD42022336617.

## 1 Introduction

Thyroid dysfunction is a common thyroid disease characterized by elevated circulating levels of thyroid hormone and thyroid-stimulating hormone (TSH). There are several types of thyroid dysfunction, including hyperthyroidism and hypothyroidism, which can be divided into overt and subclinical (1). Hyperthyroidism is a condition where thyroid hormone levels are high, with Graves’ disease (GD) as its principal cause, whereas hypothyroidism is a condition where thyroid hormone levels are low, with Hashimoto’s thyroiditis as its common cause (2). Thyroid hormones regulate several physiological processes, stimulate resting metabolic rate and thermogenesis, affect cell proliferation and development, regulate responses to other hormones, and alter carbohydrate, protein, and fat metabolism (3, 4). TSH is essential for regular function and influences metabolic rate and oxygen consumption in all tissues. Adipose tissue expresses receptors for TSH and thyroid hormones (5), and TSH may additionally be capable of amplifying adipogenesis in embryonic stem cells (6). It influences fat metabolism through a complicated interplay between the hypothalamic–pituitary–thyroid (HPT) axis and adipose tissue.

Adipose tissue no longer solely plays a passive role of energy storage; it is also a complex, essential, and dynamic metabolic and endocrine organ that produces and secretes a large amount of bioenergy molecules jointly recognized as adipokines or adipocytokines (7, 8). These adipokines include resistin, leptin, visfatin, adiponectin, and tumor necrosis factor-α (TNF-α). Adipose cytokines in the liver, skeletal muscle, and brain have endocrine, autocrine, and paracrine functions (9, 10). They regulate reproduction, immunity, thermogenesis, feeding, thyroid hormone levels, and neuroendocrine functions. As a product of adipose tissue, resistin exceeds other adipokines in regulating the reproductive system, appetite, insulin sensitivity, endocrine function, energy expenditure, immunity, bone metabolism, and inflammation (11). Moreover, it is comparable to thyroid hormones. Resistin, which was discovered in 2001 by Steppan and Lazar, is a peptide rich in cysteine through RETN gene encoding. It was called “resistin” because of the observed insulin resistance when it was injected in mice (12). Since its discovery, resistin has attracted considerable interest because of its broad range of physiological and pathological roles in several metabolic diseases. In humans, resistin is secreted with the aid of peripheral blood monocytes and other immune cells and is expressed in white adipose tissue (WAT), with the highest levels observed in female gonadal adipose tissue (12). However, in rodents, resistin is produced by adipose tissue. Simultaneously, resistin additionally has the traits of proinflammatory cytokines and is involved in insulin resistance, inflammation, and immune regulation.

Patients with thyroid dysfunction are regularly accompanied by adjustments in appetite, weight, blood lipid levels, thermogenesis, insulin resistance, and muscle mass (13). Resistin may additionally act as a bridge between thyroid dysfunction and insulin resistance (14) and may additionally interact with thyroid dysfunction in terms of inflammation and immunity. A prior animal study has demonstrated that hypothyroidism is associated with elevated resistin mRNA levels in WAT (15). However, resistin levels were severely low in mice with hyperthyroidism. Unlike in mice, human resistin is primarily derived from circulating macrophages and may additionally play a role in the inflammatory response, although its production sites are different, suggesting that human and mouse resistin can also have similar metabolic functions (16). However, in human studies exploring the association between resistin levels and thyroid dysfunction, low to high resistin levels have been reported, and their reported evidences are contradictory (14, 17–21). Therefore, the association between resistin levels and thyroid dysfunction remains controversial. The inconsistent results of these previous studies can also be due to low statistical strength, inadequate sample size, or clinical heterogeneity. However, despite resistin being a promising new diagnostic marker and potential metabolic regulator hormone, the association between resistin levels and thyroid dysfunction has not yet been elucidated. Therefore, we attempted to reconcile these disagreements and arrived at a reasonable conclusion that a systematic review and meta-analysis of the reachable records on resistin levels in patients with thyroid dysfunction is warranted. To overcome the limitations of previous studies and tackle these inconsistencies, we explored the association between resistin levels and thyroid dysfunction and analyzed the differences in resistin levels in patients with thyroid dysfunction before and after treatment.

## 2 Methods

We followed a standard protocol registered at the Centre for Reviews and Dissemination International Prospective Register of Systematic Reviews (number: CRD42022336617). This systematic review was conducted using the Preferred Reporting Items for Systematic Review and Meta-analysis statement guidelines (22).

### 2.1 Search strategy

**Figure 1:**
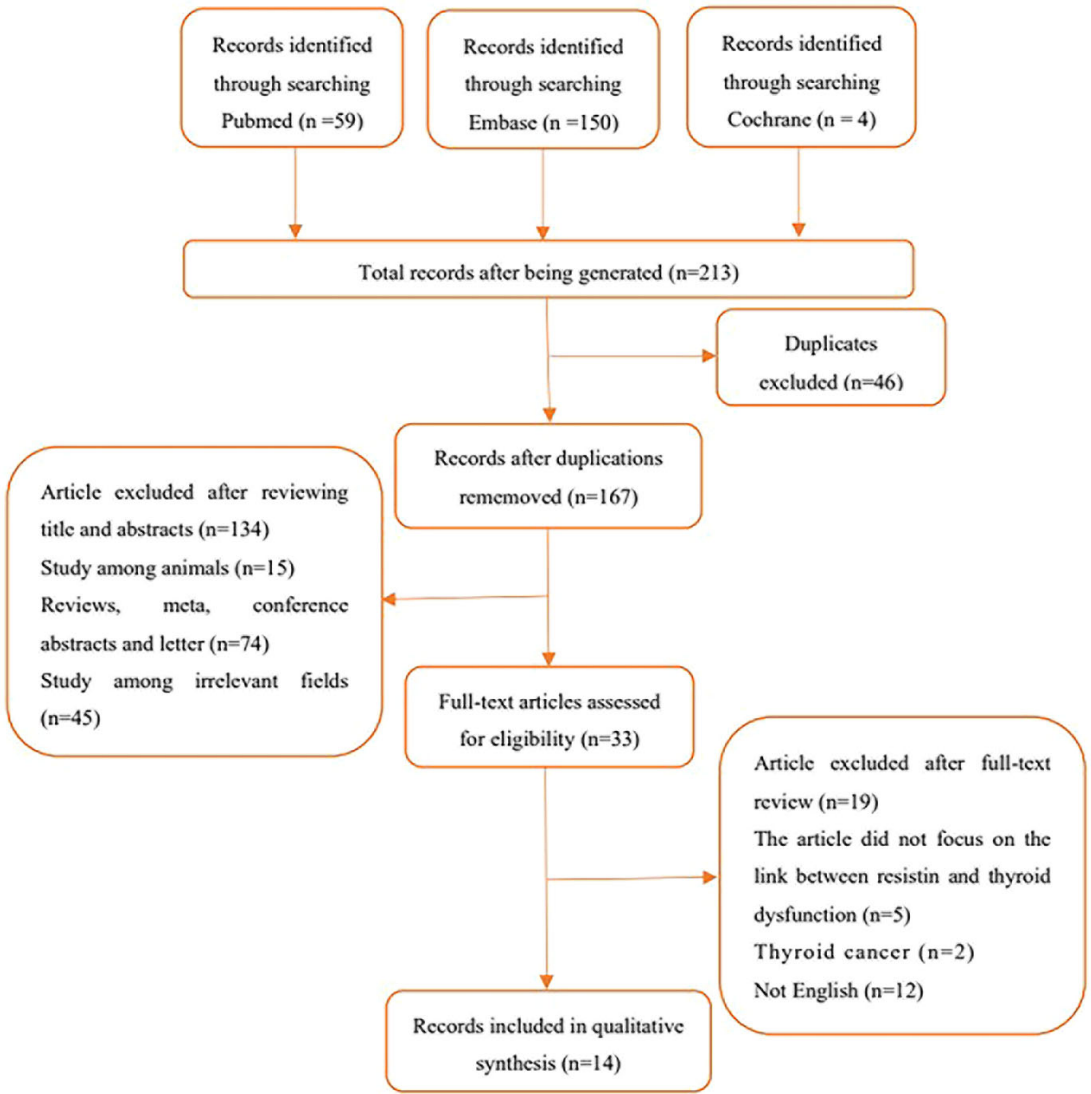
Flowchart of study selection in the meta-analysis.

To search for studies that met the inclusion criteria for this meta-analysis, studies that reported an association between serum or plasma resistin levels and thyroid dysfunction were included, particularly studies involving the comparison of resistin levels before and after treatment (surgery, drugs, radioactive iodine [^131^I]) in patients with thyroid dysfunction. The search approach was a mixture of Medical Subject Headings phrases and free terms. The Boolean logic operator AND was used to combine resistin and thyroid dysfunction terms, and the operators between the terms in these categories were concatenated with OR. Only studies written in English language and conducted in adults were considered in this study. A comprehensive search of the PubMed, Embase, and Cochrane databases was conducted until June 15, 2022, with no start date restriction. The search formula for PubMed is shown in **Table 1**, and the specific literature screening is shown in **Figure 1**.

**Table 1:** PubMed search formula and procedures.

Number	Search items
#1	("Resistin"[Mesh]) OR ((Adipocyte Cysteine-Rich Secreted Protein FIZZ3) OR (Adipocyte Cysteine Rich Secreted Protein FIZZ3))
#2	("Hypothyroidism"[Mesh]) OR ((Hypothyroidis) OR (Primary Hypothyroidism) OR (Hypothyroidism, Primary) OR (Primary Hypothyroidisms) OR (Thyroid-Stimulating Hormone Deficiency) OR (Deficiency, Thyroid-Stimulating Hormone) OR (Hormone Deficiency, Thyroid-Stimulating) OR (Thyroid Stimulating Hormone Deficiency) OR (Thyroid-Stimulating Hormone Deficiencies) OR (TSH Deficiency) OR (Deficiency, TSH) OR (TSH Deficiencies) OR (Secondary Hypothyroidism) OR (Hypothyroidism, Secondary) OR (Secondary Hypothyroidisms) OR (Central Hypothyroidism) OR (Central Hypothyroidisms) OR (Hypothyroidism, Central))
#3	("Hyperthyroidism"[Mesh]) OR ((Hyperthyroid) OR (Hyperthyroids) OR (Primary Hyperthyroidism) OR (Hyperthyroidism, Primary))
#4	("Thyroid Diseases"[Mesh]) OR ((Disease, Thyroid) OR (Diseases, Thyroid) OR (Thyroid Disease))
#5	(thyroid function) OR (thyroid diseases) OR (thyroid dysfunction) OR (thyroid disease) OR (subclinical hypothyroidism) OR (subclinical hypothyroid) OR (subclinical hyperthyroidism) OR (subclinical hyperthyroid) OR (subclinical dysthyroidism) OR (subclinical thyroid)
#6	#2 OR #3 OR #4 OR #5
#7	#1 AND #6

### 2.2 Inclusion and exclusion criteria

This systematic review focused on the association between circulating resistin levels and thyroid dysfunction or changes in resistin levels in patients with thyroid dysfunction before and after treatment. We strictly formulated the inclusion criteria based on populations, interventions, comparators, outcomes, and study designs (PICOS). The inclusion criteria were as follows: (1) observational studies assessing the association between resistin levels and thyroid dysfunction or changes in resistin levels in patients with thyroid dysfunction before and after treatment (S); (2) studies comprising participants who were adult patients with thyroid dysfunction (hyperthyroidism, hypothyroidism, subclinical hyperthyroidism, subclinical hypothyroidism) (P); (3) studies with interventions including surgery, drugs, or radioactive iodine (^131^I) (I); (4) studies where the comparator was the circulating resistin levels in the euthyroid control group or patients with thyroid dysfunction before treatment (C); and (5) studies reporting the difference in circulating resistin levels between patients with thyroid dysfunction and euthyroid controls or the difference in resistin levels before and after treatment in patients with thyroid dysfunction (O). The exclusion criteria were as follows: (1) non-original studies (review, meta-analysis, conference abstract, editorial, letter, or commentary); (2) studies comprising individuals aged < 18 years or pregnant women; (3) studies about animals, cells, tissues, or genetic variations; (4) studies with no control or raw data; (5) studies related to pathophysiological mechanisms; and (6) studies without direct or indirect data for the mean ± standard deviation (SD) of continuous variables. According to the above inclusion and exclusion criteria, the rest of the articles were reviewed to determine eligible articles. For all search results, duplicate articles were deleted, and title and abstract reviews were performed to exclude irrelevant articles. When two or more studies reported the same data, the most recently updated study was considered. Each eligible article was independently reviewed by two reviewers (Z.L. and S.K.), and these reviewers were responsible for determining which articles should be included in the final analysis. Any inconsistencies were discussed by a third investigator (L.W.)

### 2.3 Data extraction and quality assessment

Two reviewers extracted relevant information and data from all selected articles and entered them into a standard form. When a disagreement was encountered, a third reviewer resolved the disagreement, or a negotiated settlement was established. The following data were extracted: article’s research design, basic data (name of the first author, country, year of publication), sample source, number of participants, sex percentage, age, body mass index, definition of thyroid dysfunction, thyroid dysfunction type, items for detecting thyroid hormones, normal range of thyroid hormones, treatment provided, follow-up time, and test details (type of blood collection, resistin detection method, storage conditions). A formula was used to calculate the mean ± SD when the median and range or interquartile range were provided (**Table 2**).

**Table 2:** Characteristics of included studies.

First author (Year)	Country	Study design	Sample source	No	Women %	Mean age (range or SD), y		BMI		Thyroid dysfunction type	Definition of thyroid dysfunction	Normal range of thyroid hormones	Items for detecting thyroid hormones	Treatment or not	Resistin test details	Follow- up
						P	C	P	C							
Krassas et al. 2006 (23)	Greece	Prospective study	From the Endocrine Department of Panagia Hospital, Thessaloniki, Greece	83	83.1%	48.2 ± 14.6 (range, 18–73)	50.4 + 13.5 (range, 20–72)	30.5 ± 6.4	29.8 ± 5.1	Hypothyroidism	All patients with hypothyroidism had elevated TSH levels and decreased FT4 and/or TT3 levels.	TT3 (1.08–3.08 nmol/L,) FT4 (9.0–25.7 pmol/L) TSH (0.25–4.5 mIU/L) AMA (< 60U/L) ATA (< 60 U/L)	TT3, FT4, TSH	Levothyroxine treatment	Serum, EIA	4–5 months
Krassas et al. 2005 (24)	Greece	Prospective study	From the Endocrine Department of Panagia Hospital, Thessaloniki, Greece	66	66.6%	47.8 ± 13.7 (range, 19–75)	47.8 ± 11.4 (range, 24–71)	24.4 ± 4.5	25.3 ± 3.9	Hyperthyroidism	All patients with hyperthyroidism had increased TT3 and FT4 concentrations and decreased TSH concentrations	TT3 (1.08–3.08 nmol/L) FT4 (9.0–25.7 pmol/L) TSH (0.25–4.5 mIU/L)	TT3, FT4, TSH	Antithyroid drugs	Serum, EIA	3–4 months
Yaturu et al. 2004 (25)	USA	–	From the LSU Health Sciences Center and Overton Brooks VA Medical Center for hyperthyroidism	101	80.2%	42.68 ± 1.5	40 ± 2.2	25.7 ± 1.5	27.5 ± 1.0	Hyperthyroidism Hypothyroidism	–	–	FT3, FT4, TSH	Treated with radioactive iodine or antithyroid drugs	Serum, ELISA –20 °C or –80°C	–
Kaplan et al. 2012 (19)	Turkey	–	–	30	100.0%	44.0 ± 11.6	44.0 ± 11.6	29.1 ± 6.1	28.6 ± 5.9	Hypothyroidism	Euthyroidism was defined as normal serum FT4 (reference range, 0.93–1.7 ng/dL) and FT3 (reference range, 2.0–4.4 pg/mL) concentrations in association with a TSH concentration > 0.1 and < 4.0 mIU/L.	FT4 (0.93–1.7 ng/dL) FT3 (2.0–4.4 pg/mL) TSH (0.1–4.0 mIU/L)	FT3, FT4, TSH	Thyroidectomy	Serum, ELISA, −80°C	3 weeks
Iglesias et al. 2003 (17)	Spain	–	All subjects were ambulatory and were studied as outpatients during their visits to the endocrinology clinic.	60	83.3%	Hyperthyroidism (47.2 ± 3.9) Hypothyroidism (51.5 ± 4.1)	43.1± 1.8 (range, 28–78)	Hyperthyroidism (22.7 ± 0.5) Hypothyroidism (28.5 ± 1.0)	25·1 ± 0·9	Hyperthyroidism Hypothyroidism	The hyperthyroidism group showed inhibited serum TSH concentrations and high serum FT4 and T3 levels. Increased TSH concentrations associated with low levels of FT4 confirmed the presence of hypothyroidism.	–	TT3, FT4, TSH	Treated with methimazole, radioactive iodine (^131^I), subtotal thyroidectomy, levothyroxine (LT4)	Serum, ELISA, −20°C	–
Guldiken et al. 2008 (26)	Turkey	–	–	80	82.5%	Hypothyroidism (41.4 ± 8.2) Subclinical hypothyroidism (39.7 ± 10.5)	36.8 ± 5.6	Hypothyroidism (28.6 ± 5.8) Subclinical hypothyroidism (27.3 ± 4.8)	28.1± 6.8	Hypothyroidism Subclinical hypothyroidism	Hypothyroidism was defined by elevated serum thyroid-stimulating hormone (TSH) concentrations and decreased serum free T4 (FT4) concentrations. Subclinical hypothyroidism was defined as a serum TSH concentration above the upper limit of the reference associated with normal serum FT4 and FT3 concentrations.	TSH (0.4–4 μIU/mL) FT4 (0.8–1.9 ng/dL) FT3 (1.8–4.2 pg/mL)	FT3, FT4, TSH	–	Serum, ELISA	–
Gómez- Zamudio et al. 2016 (27)	México	Cross- sectional study	From the Obesity Clinic in the Hospital de Especialidades, Centro Medico Nacional Siglo XXI, IMSS in Mexico City	98	79.2%	44.1 ± 11.6	44.4 ± 12.0	47.9 ± 7.8	46.2 ± 9.1	Hypothyroidism	Hypothyroidism was considered when TSH was > 4.2 μUI/mL with FT4 < 0.93 ng/dL (overt hypothyroidism) or FT4 in normal range (subclinical hypothyroidism).	TSH (0.27–4.2 μIU/mL) FT4 (0.93–1.7 ng/dL)	FT4, TSH	–	Serum, ELISA	–
Eke Koyuncu et al. 2013 (18)	Turkey	–	–	78	78.0%	Hypothyroidism (46.53 ± 13.98) Subclinical hypothyroidism (49.06 ± 12.98) Hyperthyroidism (40.40 ± 17 .36) Subclinical hyperthyroidism (46.47 ± 13.66)	37 ± 12.53	–	–	Hypothyroidism Subclinical hypothyroidism Hyperthyroidism Subclinical hyperthyroidism	The individuals having TSH levels over 5.6 μIU/mL and serum-free T4 (fT4) levels below 0.58 ng/dL were classified as having hypothyroidism, the individuals having TSH levels over 5.6 μIU/mL and serum fT4 levels 0.58–1.64 ng/dL were classified as having subclinical hypothyroidism. The individuals having TSH levels below 0.34 μIU/mL and serum fT4 levels over 1.64 ng/dL were classified as having hyperthyroidism. The individuals having TSH levels below 0.34 μIU/mL and serum fT4 levels 0.58–1.64 ng/dL were classified as having subclinical hyperthyroidism. The patients who have TSH levels over 5.6 μIU/mL were classified as having total hypothyroidism, and the patients who have TSH levels below 0.34 μIU/mL were classified as having total hyperthyroidism.	TSH (0.34–5.6 μIU/mL) FT4 (0.58–1.64 ng/dL)	FT4, TSH	–	Serum, ELISA, −80°C	–
Dimitriadis et al. 2006 (28)	Greece	–	–	21	100.0%	45 ± 3	42 ± 4	24 ± 1	24 ± 1	Hypothyroidism	–	–	T3, T4,TSH	–	−20°C	–
Chen et al. 2016 (14)	China	Cross- sectional study	From the First Affiliated Hospital of China Medical University, Shenyang, China	782	66.2%	Hypothyroidism (46.50 ± 15.64) Hyperthyroidism (45.85 ± 13.94)	47.07 ± 15.15	Hypothyroidism (25.72 ± 2.89) Hyperthyroidism (23.76 ± 2.91)	24.55 ± 3.36	Hypothyroidism Hyperthyroidism	Patients with hyperthyroidism were diagnosed with elevated serum levels of FT3 and/or FT4 but decreased TSH levels compared to reference ranges (FT4, 9.01–19.05 pmol/L; FT3, 2.63–5.70 pmol/L; and TSH, 0.35–4.94 mIU/L). Patients with hypothyroidism were diagnosed with decreased serum levels of FT3 and/or FT4 but elevated serum levels of TSH.	FT4 (9.01–19.05pmol/L) FT3 (2.63–5.70 pmol/L) TSH (0.35–4.94 mIU/L)	FT3, FT4, TSH	–	Serum, ELISA	–
Aksoy et al. 2013 (20)	Turkey	Prospective design	–	63	100.0%	34.9 ± 10.2	33.6 ± 9.8	25.7 ± 5.2	27.2 ± 4.8	Subclinical hypothyroidism	Patients with TSH levels between 4.2 and 10 μIU/mL with normal FT4 values were considered to have subclinical hypothyroidism.	TSH (0.27–4.2 μIU/mL) FT4 (12–22 pmol/L)	FT3, FT4, TSH	L-thyroxine treatment	Serum, ELISA	6 months
Akbaba et al. 2016 (21)	Turkey	Prospective design	From the Endocrinology Clinic of Ankara Numune Training and Research Hospital.	94	77.6%	36.9 ± 10.6	34.9 ± 8.4	26.1 ± 5.5	25.7 ± 4.2	Subclinical hypothyroidism	Patients with TSH levels between 4.0 and 10 mIU/L with normal fT4 values were considered to have subclinical hypothyroidism.	–	FT3, FT4, TSH	L-thyroxin treatment.	Serum, ELISA, –20 °C	3 months
Zainab Samir Yahya Hammo et al. 2019 (8)	Iraq	Cross- sectional study	From the National Diabetes Center, which follows to Al-Mustansiriya University in Baghdad.	90	0.0%	(range, 18–68)	(range, 18–68)	–	–	Hypothyroidism Hyperthyroidism	–	–	T3, T4, TSH	–	Serum, ELISA	–
El Gawad et al. 2012 (7)	Egypt	–	From the outpatient clinics of the Specialized Medical Hospital at Mansoura University in Cairo, Egypt	70	74.3%	(range, 26–42)	(range, 25–43)	25.0 ± 1.1	27.0 ± 0.8	Hyperthyroidism	–	TSH (0.27–4.2 mIU/L) FT4 (0.93–1.7 ng/dL) FT3 (2.5–4.3 pg/mL)	FT3, FT4, TSH	Antithyroid drugs, such as carbimazole	Plasma, EIA, –70°C	3–4 months

EIA, enzyme immunoassay; ELISA, enzyme-linked immunosorbent assay; TSH, thyroid-stimulating hormone; FT3, free triiodothyronine; FT4, free thyroxine; T3, triiodothyronine; T4, thyroxine; TT_3,_ Total-triiodothyronine; AMA, antithyroid microsomal antibody; ATA, antithyroglobulin antibody; P, Patients; C, controls.

The Newcastle–Ottawa Scale (NOS) was used to determine the quality of the selected studies (29). The scale is primarily based on patient choice (up to four stars), comparability of study groups (up to two stars), and evaluation of results or exposure (up to three stars), with a total score of > 6 stars indicating excessive quality (30).

### 2.4 Statistical analyses

Meta-analysis was performed using RevMan version 5.4 and R package version 4.2.0, and P&lt;0.05 was viewed as statistically significant in a two-sided test. The weighted mean differences (MDs) and corresponding 95% confidence intervals (CIs) were calculated based on the sample size, mean, and SD extracted from eligible studies. As described by McGrath et al. (31), for studies that only provided the median and range or interquartile range of results, the mean and standard deviation were estimated using formulas. The association between resistin levels and thyroid hormones was analyzed using the meta package. P < 0.05 was considered statistically significant, indicating a correlation.

Heterogeneity between studies was assessed using Cochran’s Q statistic, with I^2^ values of 0–25%, 26–50%, 51–75%, and > 75% indicating no, low, moderate, and high heterogeneities, respectively (32). If I^2^ was < 50%, the fixed-effects model was used in the meta-analysis; otherwise, the random-effects model was used. To assess the stability of the results, we performed a sensitivity analysis by omitting one report from each rotation and recalculating the pooled estimates of the remaining studies using the Metaninf command (33). Prespecified subgroup analyses were performed to determine the main source of heterogeneity and assess the robustness of the results. Egger’s test and a visualized funnel plot were used for publication bias (34, 35), and P < 0.05 was statistically significant.

## 3 Results

### 3.1 Search results and study inclusion

In total, 213 articles were retrieved, 46 were replicated, and 134 were excluded as these articles were reviews, meta-analyses, conference abstracts, editorials, letters, animal experiments, and other unrelated areas (either not related to circulating resistin levels or thyroid disease) after title and abstract screening. The full text of the remaining 33 articles was fine-read, and the search details are shown in **Figure 1**.

### 3.2 Study characteristics

Overall, 14 studies with 1716 participants met all the inclusion criteria. Among them, six (7, 8, 14, 17, 18, 24), nine (8, 14, 17–19, 23, 26–28), four (18, 20, 21, 26), and one (18) articles were related to hyperthyroidism, hypothyroidism, subclinical hypothyroidism, and subclinical hyperthyroidism, respectively. Two studies by the same author reported an association between hyperthyroidism (2005) (24) and hypothyroidism (2006) (23) and circulating resistin levels. Therefore, both studies were included in this meta-analysis. Of the 14 studies, two (25, 27), four (17, 23, 24, 28), seven (8, 14, 18–21, 26) and one (7) were from North America, Europe, Asia, and Africa, respectively. Six studies reported follow-up times ranging from 3 weeks to 6 months. Among the included studies, 10 clarified the diagnostic criteria for hyperthyroidism and hypothyroidism; nine clarified the normal range of thyroid hormones; eight involved pre- and post-treatment comparisons of drugs, iodine-131, surgery, and other interventions; and five reported the correlation coefficient between circulating resistin levels and thyroid hormones. Enzyme immunoassay was used to detect circulating resistin levels in three studies; and enzyme-linked immunosorbent assay was used in 10 studies.

### 3.3 Overall and subgroup meta-analyses

#### 3.3.1 Association between circulating resistin levels and patients with thyroid dysfunction and euthyroid participants

**Figure 2:**
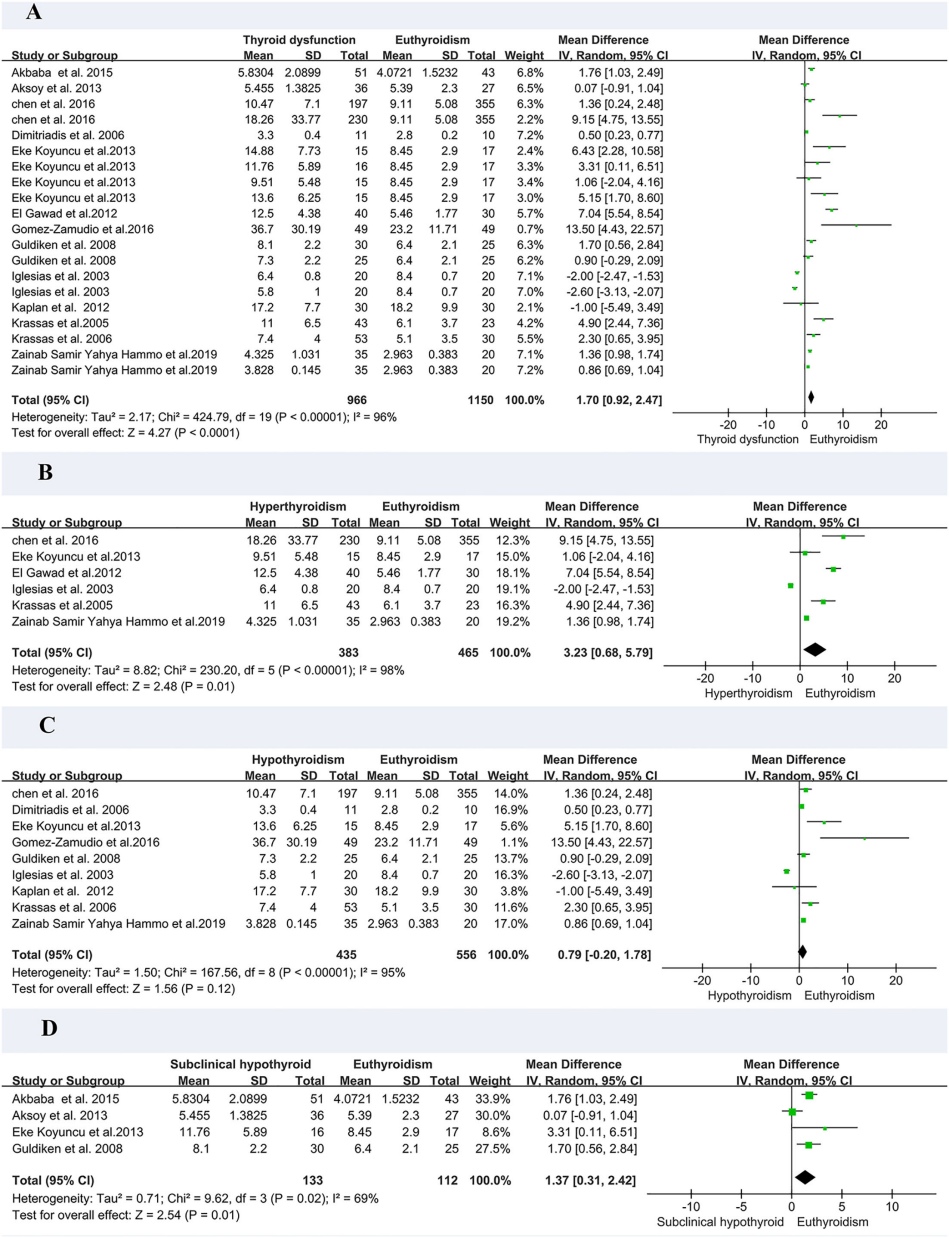
Forest plot for meta-analysis of studies on circulating resistin levels in patients with thyroid dysfunction compared with euthyroid controls **(A)**; patients with hyperthyroidism compared with euthyroid controls **(B)**; patients with hypothyroidism compared with euthyroid controls **(C)**; and patients with subclinical hypothyroidism compared with euthyroid controls **(D)**. MD, mean difference; CI, confidence interval.

**Figure 3:**
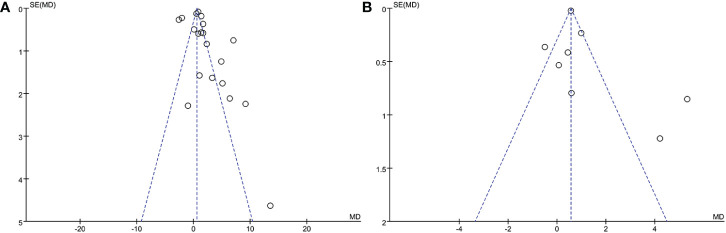
Funnel plot of included studies. **(A)** Comparison of resistin levels between patients with thyroid dysfunction and euthyroid group. **(B)** Comparison of resistin levels before and after treatment in patients with thyroid dysfunction.

In the meta-analysis of circulating resistin levels in participants with thyroid dysfunction versus euthyroid participants, I2 was > 50%; therefore, a random-effects model was used. The resistin levels of patients with thyroid dysfunction were significantly higher than those of normal controls (MD = 2.11, 95% CI = 1.11–3.11, P < 0.00001, I2 = 96%, P < 0.0001) (**Figure 2A**). Significant heterogeneity was observed between studies, requiring further analysis. Therefore, subgroup and sensitivity analyses were performed to identify the potential sources of heterogeneity. Moreover, the visual funnel (**Figure 3A**) and Egger detection (t = 1.11, P = 0.28) (**Table 3**) showed no heterogeneity.

**Table 3:** Publication bias-Egger’s tests.

	Publication bias - Egger’s tests	
	t value	P value
Total hyperthyroidism: hyperthyroidism and subclinical hyperthyroidism	t = 0.99	P = 0.37
Hyperthyroidism	t = 1.07	P = 0.35
Total hypothyroidism: hypothyroidism and subclinical hypothyroidism	t = 0.38	P = 0.71
Hypothyroidism	t = 0.01	P > 0.99
Total thyroid dysfunction	t = 1.11	P = 0.28
Thyroid dysfunction before and after treatment	t = 0.89	P = 0.41

As there was only one study on subclinical hyperthyroidism, we performed a subgroup analysis of hyperthyroidism, hypothyroidism, and subclinical hyperthyroidism. A meta-analysis of six studies involving circulating resistin levels between patients with hyperthyroidism and euthyroid controls demonstrated that the resistin levels of patients with hyperthyroidism were significantly higher than those of euthyroid controls (MD = 3.23, 95% CI = 0.68–5.79, P = 0.01) (**Figure 2B**). A meta-analysis of nine studies demonstrated that there was no difference in resistin levels between patients with hypothyroidism and euthyroid controls (MD = 0.79, 95% CI = –0.20–1.78, P = 0.12) (**Figure 2C**). A meta-analysis of four studies confirmed that the resistin levels of patients with subclinical hypothyroidism were significantly higher than those of euthyroid controls (MD = 1.37, 95% CI = 0.31–2.42, P < 0.05, I2 = 69%, Pheterogeneity < 0.05) (**Figure 2D**).

#### 3.3.2 Comparison of resistin levels before and after treatment of thyroid dysfunction

**Figure 4:**
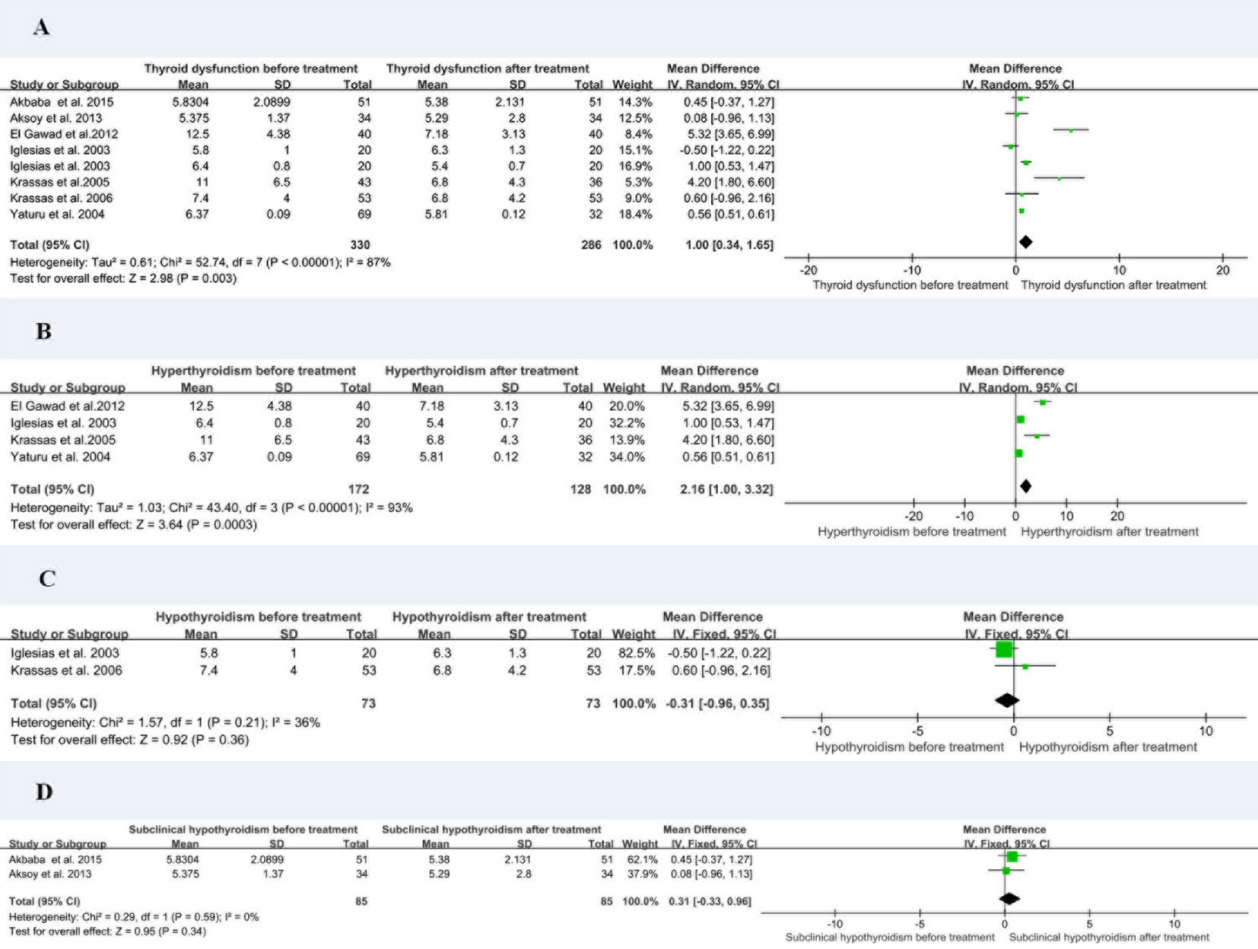
Forest plot for meta-analysis of circulating resistin levels in patients with thyroid dysfunction before and after treatment **(A)**; patients with hyperthyroidism before and after treatment **(B)**; patients with hypothyroidism before and after treatment **(C)**; and patients with subclinical hypothyroidism before and after treatment **(D)**. MD, mean difference; CI, confidence interval.

Eight of the studies reported resistin levels in patients with thyroid dysfunction before and after treatment with antithyroid drugs, iodine-131, and surgery. The duration of treatment follow-up ranged from 3 weeks to 6 months, and most patients returned to a normal thyroid status. The meta-analysis showed an I2 value of 87% in patients with thyroid dysfunction before and after treatment. The random-effects model demonstrated that the resistin levels after treatment were significantly lower than those before treatment (MD = 1.00, 95% CI = 0.34–1.65, P < 0.05, I2 = 87%, Pheterogeneity < 0.00001) (**Figure 4A**). I2 value at 87% indicated large heterogeneity. Subgroup and sensitivity analyses were performed to verify the stability of the results of the meta-analysis. Meanwhile, the visual funnel plot (**Figure 3B**) and Egger’s test (t = 0.89, P = 0.41) (**Table 3**) indicated that there was no heterogeneity.

The eight studies were divided into hyperthyroidism, hypothyroidism, and subclinical hypothyroidism before and after treatment for subgroup analysis. The resistin levels of patients with hyperthyroidism after treatment were significantly lower than those before treatment (MD = 2.16, 95% CI = 1.00–3.32, P < 0.05, I2 = 93%, Pheterogeneity = 0.0003) (**Figure 4B**). There was no substantial difference in resistin levels between patients with hypothyroidism (MD = –0.31, 95% CI = –0.96–0.35, P > 0.05, I2 = 36%) and subclinical hypothyroidism (MD = 0.31, 95% CI = –0.33–0.96, P > 0.05, I2 = 0%) before and after treatment (**Figures 4C, D**).

### 3.4 Sensitivity analyses

**Figure 5:**
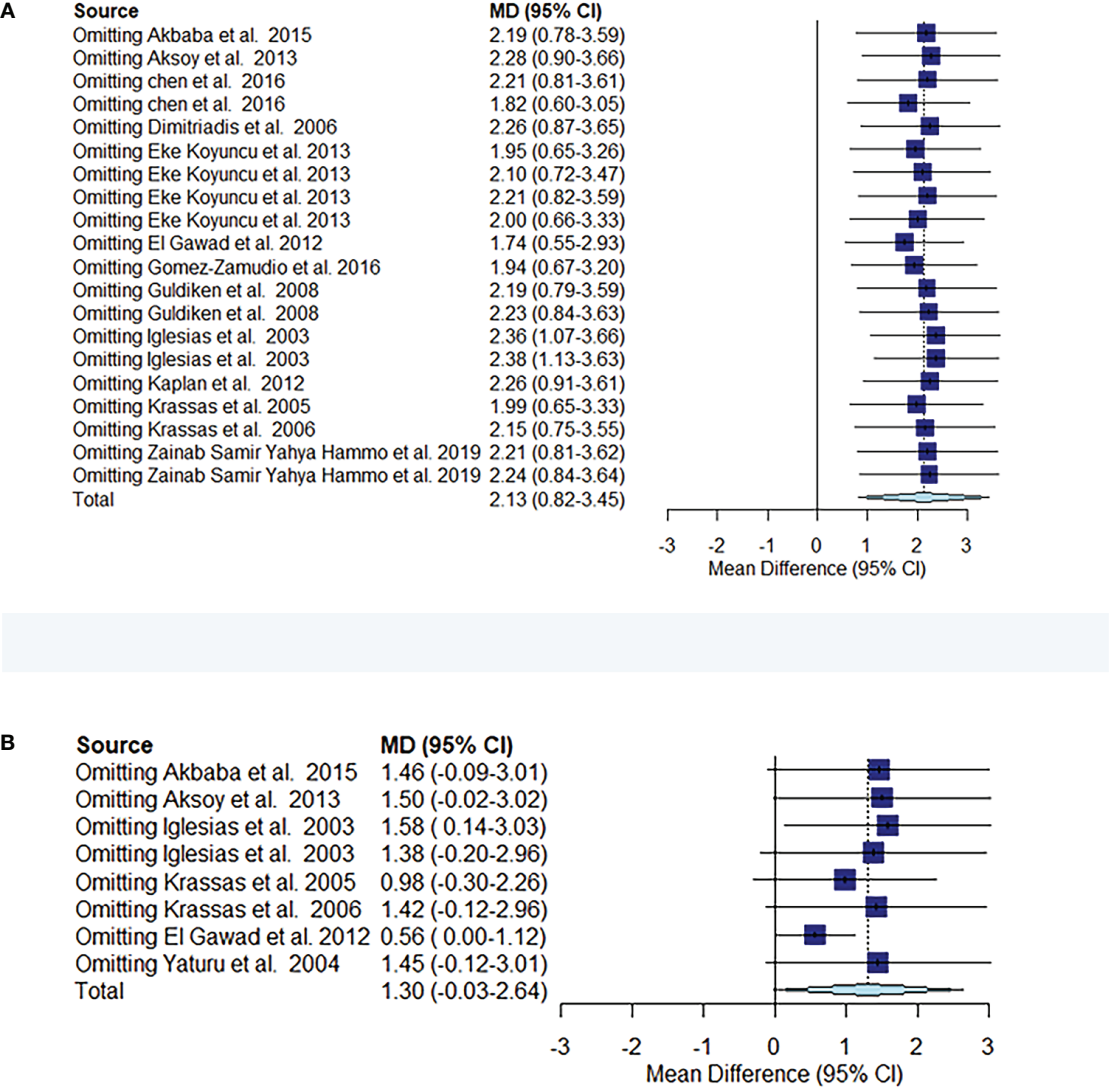
Meta-analysis estimates, with given named study being omitted. Sensitivity analysis of circulating resistin levels between patients with thyroid dysfunction and euthyroid controls **(A)**. Sensitivity analysis of circulating resistin levels in patients with thyroid dysfunction before and after treatment **(B)**.

The comparison of circulating resistin levels between patients with thyroid dysfunction and euthyroid participants before and after treatment for thyroid dysfunction showed heterogeneity, with I2 values of 96% and 87%, respectively. Further sensitivity analyses were performed to determine potential sources of heterogeneity and demonstrated that the overall statistical significance did not change when any of the studies were omitted. Hence, the results of this meta-analysis were considered relatively credible (**Figures 5A, B**).

### 3.5 Publication bias

The visual funnel plot (**Figure 3**) processing and publication bias Egger’s test (**Table 3**) were performed to compare circulating resistin levels between patients with thyroid dysfunction and euthyroid participants and before and after treatment for thyroid dysfunction. This aimed to identify potential publication bias in meta-analyses of the included studies (≥ 5). All the P-values of Egger’s test were > 0.05. All of these effects indicated that there was no publication bias.

### 3.6 Correlation between resistin levels and thyroid hormone levels

Five studies reported a correlation between resistin levels and thyroid hormone levels, including TSH, free triiodothyronine (FT3), free thyroxine (FT4), T3, and T4. The Metaphor package was used to summarize the results, which showed a positive correlation between resistin levels and FT3 levels in thyroid dysfunction (r = 0.275, P = 0.001) (**Table 4**).

**Table 4:** Correlation between resistin levels and thyroid hormone levels in patients with thyroid dysfunction.

Thyroid hormone	N(groups)-n(number)	r	95%CI	I^2^	P value for heterogeneity	P value
FT3	2-141	0.28	0.11, 0.42	0.00%	0.62	0.001
FT4	2-141	0.24	-0.07, 0.51	65.47%	0.09	0.12
TSH	8-308	0.28	-0.02, 0.53	83.76%	< .00001	0.07
T3	2-70	-0.10	-0.39, 0.20	34.85%	0.21	0.49
T4	2-70	-0.05	-0.29, 0.19	0.00%	0.50	0.67

TSH , thyroid-stimulating hormone; FT3, free triiodothyronine; FT4, free thyroxine; T3, triiodothyronine; T4, thyroxine; CI, confidence interval; r, correlation coefficient.

### 3.7 Assessment of the quality of the included studies

The NOS was used to assess the quality of the included observational studies (**Table 5**). Ten studies were of high quality and appropriately defined thyroid dysfunction. Only one study (Dimitriadis et al., 2006) did not specify the source of the sample and had no definition of thyroid dysfunction, no normal reference range for thyroid hormones, no address method of resistance extraction, and no follow-up information. The NOS score was 5.

**Table 5:** Newcastle-Ottawa score of the included studies.

Quality indicators from Newcastle-Ottawa Scale	Selection	Comparability	Outcome/exposure	Total
Krassas et al. 2006 (23)	4	2	3	9
Krassas et al. 2005 (24)	4	2	2	8
Yaturu et al. 2004 (25)	3	2	1	6
Kaplan et al. 2012 (19)	4	2	2	8
Iglesias et al. 2003 (17)	3	2	1	6
Guldiken et al. 2008 (26)	4	2	1	7
Gómez-Zamudio et al. 2016 (27)	4	2	1	7
Eke Koyuncu et al. 2013 (18)	4	2	1	7
Dimitriadis et al. 2006 (28)	3	2	0	5
Chen et al. 2016 (14)	4	2	1	7
Aksoy et al. 2013 (20)	4	2	2	8
Akbaba et al. 2016 (21)	3	2	3	8
Zainab Samir Yahya Hammo et al. 2019 (8)	3	2	1	6
El Gawad et al. 2012 (7)	3	2	3	8

### 3.8 Another subgroup

Additional subgroup analyses were performed to investigate potential sources of heterogeneity (**Table 6**). Notably, some observational studies had relatively small sample sizes, which may have contributed to the lack of statistical power in subgroup analyses. Subgroup analysis was performed on study location (Asia, Europe, North America, and Africa), study design (cross-sectional, prospective study, not explained), sex (male, female, male and female comparison), quality score (≤ 6, > 6), presence of follow-up, and homeostasis model assessment-estimated insulin resistance (HOMA-IR) score (≤ 2.50, > 2.50).

**Table 6:** Another subgroup analysis of included studies.

Stratification groups	Data Points (N)	Cases		Random / Fix - effects MD (95% CI)	P - value	Heterogeneity	
		Thyroid dysfunction	Controls			I^2^ (%)	P - value
**All studies**	20	966	1150	1.70 (0.92, 2.47)	Z = 4.27 ( P < 0.0001)	96%	< 0.0001
**Before and after treatment**	8	330	286	1.00 (0.34, 1.65)	Z = 2.98 ( P = 0.003)	87%	< 0.00001
Study location							
Asian	13	730	968	1.44 (0.91, 1.97)	Z = 5.32 ( P < 0.00001)	72%	< 0.0001
Europe	5	147	103	0.30 (−1.48, 2.08)	Z = 0.33 ( P = 0.74)	98%	< 0.00001
Other regions: North America, Africa	2	89	79	7.21 (5.73, 8.69)	Z = 9.56 ( P < 0.00001)	47%	0.17
Asian - after treatment	2	85	85	0.31 (−0.33, 0.96)	Z = 0.95 ( P = 0.34)	0%	0.59
Europe - after treatment	4	136	129	0.95 (−0.33, 2.22)	Z = 1.46 ( P = 0.15)	85%	0.0001
Other areas - after treatment	2	109	72	2.86 (−1.80, 7.53)	Z = 1.20 ( P = 0.23)	97%	< 0.00001
Study design							
Cross - sectional study	5	546	799	1.52 (0.70, 2.34)	Z = 3.62 ( P = 0.0003)	85%	< 0.0001
Prospective study	4	183	123	1.94 (0.45, 3.43)	Z = 2.55 ( P = 0.01)	82%	0.0007
Unspecified	11	237	228	1.56 (0.09, 3.03)	Z = 2.09 ( P = 0.04)	97%	< 0.00001
Prospective study - after treatment	4	181	174	0.93 (−0.24, 2.1)	Z = 1.56 ( P = 0.12)	69%	0.02
Unspecified - after treatment	4	149	112	1.15 (0.21, 2.10)	Z = 2.39 ( P = 0.02)	93%	< 0.00001
Gender							
man	5	88	113	0.62 (−0.25, 1.48)	Z = 1.40 ( P = 0.16)	86%	< 0.00001
woman	6	168	140	0.48 (−1.22, 2.17)	Z = 0.55 ( P = 0.58)	95%	< 0.00001
Comparing men and women	3	22	107	1.45 (−1.27, 4.17)	Z = 1.05 ( P = 0.30)	88%	< 0.0001
Men - after treatment	3	18	18	−1.02 (−5.76, 3.72)	Z = 0.42 ( P = 0.67)	79%	0.009
Women - after treatment	4	121	121	0.95 (−0.60, 2.51)	Z = 1.20 ( P = 0.23)	58%	0.07
Comparison of men and women - after treatment	4	22	107	3.57 (2.13, 5.00)	Z = 4.87 ( P < 0.00001)	62%	0.05
Quality score							
> 6	15	845	1060	3.00 (1.81, 4.19)	Z = 4.93 ( P < 0.00001)	85%	< 0.00001
≤ 6	5	121	90	−0.36 (−1.53, 0.82)	Z = 0.59 ( P = 0.55)	99%	< 0.00001
> 6 - after treatment	5	221	214	1.98 (0.15, 3.80)	Z = 2.12 ( P = 0.03)	89%	< 0.00001
≤ 6 - after treatment	3	109	72	0.44 (−0.13, 1.00)	Z = 1.52 ( P = 0.13)	83%	0.003
Resistin levels							
< 14.8 ng/ml	16	642	699	1.36 (0.57, 2.15)	Z = 3.39 ( P = 0.0007)	96%	< 0.00001
≥ 14.8 ng/ml	4	324	451	6.38 (0.91, 11.85)	Z = 2.29 ( P = 0.02)	78%	0.003
Follow - up							
Yes	6	253	183	2.68 (0.56, 4.80)	Z = 2.48 ( P = 0.01)	92%	< 0.00001
No / unspecified	14	713	967	1.16 (0.28, 2.04)	Z = 2.59 ( P = 0.010)	96%	< 0.00001
Yes - after treatment	5	221	214	1.98 (0.15, 3.80)	Z = 2.12 ( P = 0.03)	89%	< 0.00001
No - after treatment	3	109	72	0.44 (−0.13, 1.00)	Z = 1.52 ( P = 0.13)	83%	0.003
HOMA - IR							
> 2.50	8	639	889	2.3 (−0.01, 4.61)	Z = 1.95 ( P = 0.05)	97%	< 0.00001
≤ 2.50	4	142	120	1.13 (0.30, 1.96)	Z = 2.66 ( P = 0.008)	64%	0.04
Unspecified	8	185	141	1.36 (0.79, 1.94)	Z = 4.67 ( P < 0.00001)	82%	< 0.00001
> 2.50 - after treatment	4	133	133	1.46 (−0.22, 3.15)	Z = 1.70 ( P = 0.09)	93%	< 0.00001
≤ 2.50 - after treatment	2	85	85	0.31 (−0.33, 0.96)	Z = 0.95 ( P = 0.34)	0%	0.59
Unspecified - after treatment	2	112	68	2.17 (−1.37, 5.72)	Z = 1.20 ( P = 0.23)	89%	0.003

MD, mean difference; CI, confidence interval.

In our study, unexpectedly, resistin levels were significantly higher in men than in women after treatment (MD = 3.57, 95% CI = 2.13–5.00, P < 0.00001, I2 = 62%, P_heterogeneity_ = 0.05). When the cutoff point of HOMA-IR was 2.50, the resistin levels of patients with thyroid dysfunction in the low HOMA-IR group were significantly higher than those of euthyroid participants (MD = 1.13, 95% CI = 0.30–1.96, P = 0.008, I2 = 64%, P_heterogeneity_ = 0.04). Nevertheless, because of the small number of included studies, reliability should be further considered.

## 4 Discussion

To statistically and quantitatively test the association between resistin levels and thyroid dysfunction, 14 articles that were searched in online databases met our inclusion criteria. The expression of thyroid dysfunction can be clinical or subclinical, depending on the extent of the disrupted thyroid parenchyma. Hyperthyroidism is characterized by a TSH level < 0.45 mIU/L and an FT4 level higher than the reference range. Subclinical hyperthyroidism is characterized by a TSH level < 0.45 mIU/L and an FT4 level within the reference range, or only < 0.45 mIU/L when FT4 level is not measured. Clinical hypothyroidism is a common hormone deficiency disease characterized by a TSH level ≥ 20 mIU/L or a TSH level ≥ 4.50 mIU/L, with an FT4 level below the reference range. Subclinical hypothyroidism is characterized by a TSH level of 4.50–20 mIU/L and an FT4 level within the reference range (36). The results of the overall analysis confirmed that resistin levels were considerably higher in patients with thyroid dysfunction than in euthyroid controls, suggesting that resistin levels may be significantly associated with thyroid dysfunction. Patients with thyroid dysfunction had significantly lower resistin levels after treatment than before treatment, although there was extensive heterogeneity among studies. The resistin levels of men with thyroid dysfunction were significantly higher than those of women with thyroid dysfunction after treatment, and the resistin levels of patients with thyroid dysfunction in the low HOMA-IR group were significantly higher than those of the euthyroid controls. However, owing to the small number of included studies, reliability should be further considered. No publication bias was observed in this meta-analysis. Considering the association between resistin levels and thyroid dysfunction and inconsistent published results in this context, the present meta-analysis is of significant value.

This meta-analysis demonstrated that when thyroid dysfunction was analyzed by subgroups of hyperthyroidism, hypothyroidism, and subclinical hypothyroidism (subclinical hyperthyroidism was not analyzed because only one study was involved), patients with hyperthyroidism and subclinical hypothyroidism had higher resistin levels than euthyroid controls. However, the association between resistin levels and hypothyroidism remains unclear. After treatment with iodine-131, surgery, and other interventions, resistin levels decreased significantly. However, no sizeable differences were observed between hypothyroidism and subclinical hypothyroidism. In addition, sex differences between resistin levels and thyroid dysfunction were not mentioned in most of the studies included in our meta-analysis. Our summary of studies involving sex showed that men with thyroid dysfunction had significantly higher resistin levels than women with thyroid dysfunction after treatment. In animal experiments, the expression of resistin in rats showed evident sexual dimorphism, and the expression level of the resistin gene in male rats was higher than that in female rats, which is similar to our study (15). In addition, the results of the correlation analysis between thyroid hormone levels and resistin levels confirmed that resistin levels were positively correlated with FT3 levels (r = 0.27578, P = 0.001). This provides evidence for the association between resistin levels and thyroid function. We covered only a limited number of studies in Asian, European, North American, and African countries. Additionally, this finding may not mirror the association between resistin levels and thyroid dysfunction in different ethnic groups. In the included studies, clear diagnostic criteria and thyroid hormone reference ranges ensured the reliability of our analysis to some extent. Changes in thyroid hormone levels may affect resistin synthesis and/or secretion in adipose tissue and/or macrophages (18).

Since resistin was discovered, various studies have investigated its association with several metabolic diseases, such as obesity, metabolic syndrome, insulin resistance, diabetes, and other related diseases (37). Resistin, a 12.5 kDa polypeptide encoded by the human RETN gene, is still being explored for its potential role as a therapeutic and diagnostic target in several metabolic diseases (38). Individuals with hyperthyroidism have higher resistin levels, and excess thyroid hormone levels induce insulin resistance in the liver and surrounding tissues (17). Resistin may also be associated with insulin resistance and thyroid dysfunction. It also suggests a possible association between HPT axis function and resistin levels (14). Insulin resistance can be divided into peripheral and hepatic types. Hypothyroidism is usually characterized by peripheral insulin resistance in the skeletal muscle and adipose tissue, whereas in hyperthyroidism, both hepatic and peripheral insulin resistance are observed (14, 39–41). However, our study found no association between thyroid dysfunction and hyperinsulinism resistance. When we stratified the analysis of HOMA-IR with a cutoff point of 2.50 (42, 43), resistin levels were drastically higher in patients with thyroid dysfunction than in euthyroid controls solely in the low HOMA-IR group. Some researchers have suggested that insulin is an inhibitor of resistin, but its complicated mechanism requires further exploration (44). Although the function of resistin in thyroid dysfunction remains to be elucidated, several mechanisms can be considered. Resistin has some characteristics of proinflammatory cytokines and plays a role in inflammation. Resistin produces proinflammatory cytokines, including interleukin (IL)-12, IL-6, IL-1β, and TNF-α, through the activation of TLR4 receptor stimulation and proinflammatory effects mediated by the traditional nuclear factor kappa B pathway (45, 46). These cytokines further enhance the expression of resistin and may directly affect the pathogenesis of thyroid dysfunction, forming a pathogenic cycle. A prior animal study has also demonstrated that mice can produce human resistin to improve WAT inflammation and insulin resistance under specific conditions stimulated by a high-fat diet (47). Interestingly, increased resistin levels are closely associated with rheumatoid arthritis, systemic lupus erythematosus, psoriasis, and other autoimmune diseases, suggesting that resistin may be a useful marker of systemic inflammatory status in autoimmune diseases (45, 48). However, its expression may decrease after treatment or remission. Thyroid dysfunction often includes hypothyroidism and hyperthyroidism and is generally caused by autoimmune thyroid diseases (AITDs), such as GD or Hashimoto’s thyroiditis. The etiology of resistin and AITD is complex, involving both genetic and environmental factors (2). Therefore, we hypothesize that there may be an association between autoimmune factors and resistin levels in the occurrence and development of thyroid dysfunction. A limitation of our study is that we did not include studies assessing the association between resistin levels and thyroid autoantibodies, and further studies are required to determine the exact association between the pathogenesis of thyroid dysfunction and resistin levels.

An excessive heterogeneity was once determined in these analyses, and variations in clinical presentation might have also contributed to the heterogeneity. Resistin secretion by macrophages outside human adipose tissue, differences in individual inflammatory status, patient sex, treatment changes, antibody concentrations, metabolic effects of other hormones, and intermediate metabolism may have potential effects. Heterogeneity in study design, short follow-up time, presence of drugs that affect patients’ lipid profiles, differences in circulating resistin storage conditions and detection methods, and thyroid dysfunction (degree, duration, and cause) may play a role. A set of thyroid hormone levels may lead to misdiagnosis of transient subclinical thyroid dysfunction. The means and standard deviations estimated from the median, first percentile, and third percentile are also biased. Simultaneously, the demographic characteristics and characteristics of the study population need to be explored in the future. Some limitations of this study include the small number of included studies exploring the association between resistin levels and subclinical hyperthyroidism and sex differences between resistin levels and thyroid dysfunction, making it difficult to extrapolate conclusions to different populations worldwide. Therefore, further cohort studies should be conducted to characterize the potential causal association between thyroid dysfunction and resistin levels.

To the best of our knowledge, this is the first meta-analysis to assess circulating resistin levels in patients with thyroid dysfunction, and inconsistent results were rigorously quantified and analyzed, leading to more robust conclusions. Most of the covered studies were based entirely on the high quality of the NOS scoring system, which ensured the credibility of our results. Notably, the quantity of the selected studies with eligible data used to be small; thus, it must be carefully interpreted. Due to language limitations, our study did not have access to all available sources reporting data related to resistin levels and thyroid dysfunction. Some articles not written in English language were not included in our study, and several countries are not native speakers of English. Thus, we may have missed some suitable articles written in other languages. Therefore, larger clinical trials are required to validate these results.

## 5 Conclusion

In conclusion, this meta-analysis revealed an association between thyroid dysfunction and resistin levels. This meta-analysis confirms that resistin is significantly associated with an increased risk of hyperthyroidism and subclinical hypothyroidism, but it has only a small effect on hypothyroidism. The circulating resistin levels of patients with hyperthyroidism decrease significantly after treatment, and there is no difference in resistin levels before and after treatment for patients with hypothyroidism and subclinical hypothyroidism. The results of this meta-analysis suggest that resistin may play a role in insulin resistance, inflammation, and immunology in the pathogenesis of thyroid dysfunction. Resistin may be a potential marker of thyroid dysfunction and an effective therapeutic target. Future studies should focus on enrolling more ethnically diverse patients and those with subclinical thyroid dysfunction. If conditions permit, resistin level can be used as a valuable biomarker to assess the clinical status of thyroid dysfunction. Given the above limitations, more large-scale, well-designed randomized and experimental studies are required to confirm the effect of resistin levels on the development of thyroid dysfunction in the future.

## Data availability statement

The original contributions presented in the study are included in the article/**Supplementary Material**. Further inquiries can be directed to the corresponding author.

## Author contributions

Conceptualization: ZL. Methodology (data collection): ZL and SK. Statistical analyses: ZL, SK, and LW. Writing (original draft preparation): ZL and SK. Review and editing: LW. All authors have contributed to the manuscript and approved the submitted version.
